# Improving UWB-Based Localization in IoT Scenarios with Statistical Models of Distance Error

**DOI:** 10.3390/s18051592

**Published:** 2018-05-17

**Authors:** Stefania Monica, Gianluigi Ferrari

**Affiliations:** 1 Department of Mathematics, Physics and Computer Science, University of Parma, 43124 Parma, Italy; stefania.monica@unipr.it; 2 Department of Engineering and Architecture, University of Parma, 43124 Parma, Italy

**Keywords:** ultra wide band, experimental model, indoor localization, Internet of Things, least square method

## Abstract

Interest in the Internet of Things (IoT) is rapidly increasing, as the number of connected devices is exponentially growing. One of the application scenarios envisaged for IoT technologies involves indoor localization and context awareness. In this paper, we focus on a localization approach that relies on a particular type of communication technology, namely Ultra Wide Band (UWB). UWB technology is an attractive choice for indoor localization, owing to its high accuracy. Since localization algorithms typically rely on estimated inter-node distances, the goal of this paper is to evaluate the improvement brought by a simple (linear) statistical model of the distance error. On the basis of an extensive experimental measurement campaign, we propose a general analytical framework, based on a Least Square (LS) method, to derive a novel statistical model for the range estimation error between a pair of UWB nodes. The proposed statistical model is then applied to improve the performance of a few illustrative localization algorithms in various realistic scenarios. The obtained experimental results show that the use of the proposed statistical model improves the accuracy of the considered localization algorithms with a reduction of the localization error up to 66%.

## 1. Introduction

Nowadays, the number of interconnected devices is highly increasing and it is expected that in the near future it will continue its exponential growth. For this reason, Internet of Things (IoT) is a very up-to-date research topic, which involves various application scenarios, with the aim of developing smart environments [1]. One of the enabling technologies related to IoT is localization, which can be considered the estimation problem of locating objects and users from radio signals, in order to provide context aware services to users. As possible applications of location-based services, it is worth mentioning: facilitating navigation inside large indoor areas, such as shopping mall, airports, and train stations; tracking people and objects inside buildings; and locating goods stored inside warehouses. All of these scenarios can be considered as related to IoT applications. In all such cases, the Global Positioning System (GPS) cannot be used, as it is not helpful in indoor environments. Therefore, in recent years, different technologies have been proposed to solve indoor localization issues [2]. In this paper, we focus on one of these technologies, namely Ultra Wide Band (UWB), which can be fruitfully applied in IoT scenarios.

UWB technology is very attractive for indoor communications. The large bandwidth and the high time resolution of UWB signals can theoretically reduce the impact of phenomena, which typically interfere with wireless communications in indoor environments (e.g., non-line-of-sight propagation, multipath, and multiple access interference) [3]. As a matter of fact, the large frequency spectrum, which characterizes UWB signals, guarantees the capability of penetrating through obstacles. Moreover, UWB systems transmit very short duration pulses, usually on the order of nanoseconds, with a low duty cycle. On one hand, this guarantees accurate Time of Flight (ToF) estimation of signals traveling between nodes, reducing the impact of negative issues such as non-line-of-sight propagation; on the other hand, it guarantees low energy consumption. Moreover, since UWB signals have very large bandwidth, UWB systems have low transmit power in order to avoid interference problems with other devices operating in the same frequency spectrum [3].

In the late 1980s, UWB signals were first used for military applications. The interest in UWB communications grew after the Federal Communications Commission (FCC) in the USA allowed the unlicensed use of UWB devices in February 2002 and after the definition, in 2007, of the IEEE 802.15.4a standard, which provides physical layer specifications for short-range low data-rate UWB-based communications [4]. Nowadays, UWB systems represent a promising technology in many fields, such as: assisted living [5]; security area surveillance [6]; gaming [7]; localization and tracking of people, vehicles, and goods [8,9]. As a consequence, a few commercial products are available. It is worth noting that UWB cannot still be considered as ubiquitous, but its attraction is increasing and there are also some mobile devices, such as smartphones, which include such a technology.

One of the most promising aspects of UWB radios is their potential for high-precision localization, making them attractive for Wireless Sensor Network (WSN) scenarios. More precisely, positioning techniques for WSNs can be divided into two classes: range-free and range-based [10]. As indicated by their name, *range-free approaches* do not require the availability of range measurements between nodes. These techniques can be further divided into local techniques, such as the centroid algorithm [11] or the Approximate Point in Triangulation (APIT) algorithm [10], and hop-counting techniques, such as the Distance Vector Hop (DV-Hop) algorithm [12].

At the opposite, *range-based approaches* rely on the estimation of a signal parameter, such as the ToF or the Received Signal Strength (RSS) [13]. The use of range-based approaches is particularly suitable when dealing with the UWB technology. In particular, time-based positioning techniques, which rely on measurements of the ToF of signals travelling between nodes, are typically preferred—as a matter of fact, as intuitively expected, the accuracy of time-based approaches improves as the bandwidth of the signal increases [14]. Given the ToF of a signal traveling between a pair of nodes, the distance between them can be directly obtained by multiplying the ToF by the propagation speed.

In this paper, on the basis of an extensive experimental campaign with PulsON 410 Ranging and Communications Modules (P410 RCMs) produced by Time Domain.

Ref [15], we derive a statistical model for the range estimation error between two P410. Then, we test the validity of the considered statistical model on realistic localization problems that involve the use of four RCMs (three anchor nodes and one target node). In particular, the range estimation error model is applied to improve the performance of two existing localization algorithms, namely the Circumference Intersection (CI) algorithm [16] and the Two-Stage Maximum-Likelihood (TSML) algorithm [17]. Such algorithms have already been applied in a different localization scenario and without the statistical model proposed in this paper in [18]. Both algorithms are then applied in two illustrative localization scenarios, namely, with “good” and “bad” nodes’ topologies. Our results show that the statistical model derived in the first part of the paper can significantly improve the accuracy of the considered localization algorithms, with a reduction of the position estimation error up to 66%. In [19], a swarm-based algorithm is used to address localization and a statistical model similar (but different from) to that proposed in this paper is used.

The paper is organized as follows. In Section 2, the reference experimental setup is first described and, then, the statistical model for the range estimation error, based on the introduced setup, is derived. In Section 3, the general localization problem is introduced and two (known) localization algorithms are described. In Section 4, two localization scenarios are considered and the improvement, brought by the application of the statistical model of the range estimation error introduced in Section 2 to the localization algorithms considered in Section 3, is quantified. Finally, Section 5 concludes the paper.

## 2. Statistical Model of the Range Estimation Error: Experimental Derivation

**Figure 1:**
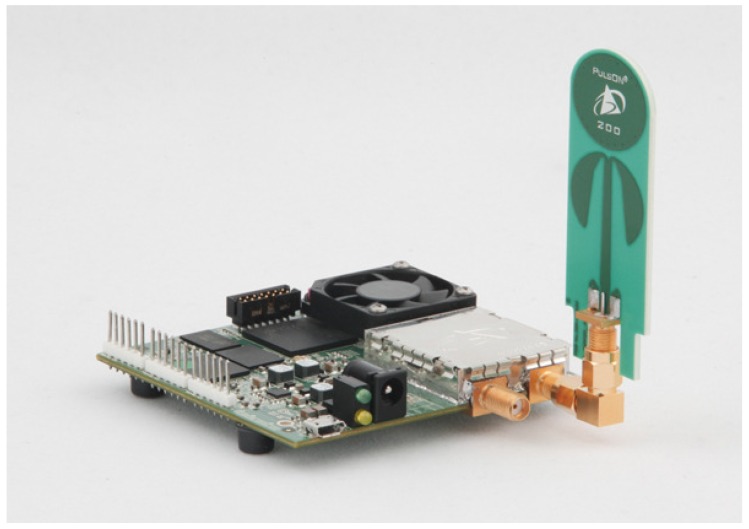
A P410 RCM used to obtain experimental results.

In order to derive an analytical model for the range estimation error, an extensive experimental campaign of range measurements between two pairs of UWB sensors is carried out. The UWB sensors used to collect our results are the P410, which are equipped with RCM firmware and software (version 2.1 build 3, Time Domain, Huntsville, AL, USA) to enable ranging purposes. One of the used boards is shown in Figure 1.

Such sensors are single-board UWB radio components that can be integrated into users’ electronic devices to obtain high precision range measurement. The Radio Frequency (RF) transmission of the RCMs is centered at 4.3 GHz and a bandwidth of more than 1 GHz is guaranteed, as the RF band is from 3.1 GHz to 5.3 GHz. Each sensor is composed of a single board with dimensions 7.6×8.0×1.6 cm${}^{3}$ and a UWB antenna. Each sensor needs to be powered. The RCMs can be programmed using C or Matlab libraries (version 2.1, Time Domain, Huntsville, AL, U.S.A.) provided by Time Domain. The software version of the RCMs used in this work is 2.4. The transmit power is −14.5 dBm and the value of the Pulse Integration Index (PII) has been set equal to 5: this corresponds to a data rate of 316 kpbs. Moreover, range estimates are obtained with a frequency of 118 Hz. Range estimates are defined as the Precision Range Measurements (PRMs), namely the two-way ToF distance between the requesting and responding RCMs, which is returned in millimeters [15]. A typical UWB communication architecture involves many RCMs, with corresponding assigned IDs, and at least one host, connected to a RCM via USB connection. The communication between pairs of RCMs can be carried out via broadcast or by directly interrogating a given ID corresponding to a RCM. The requests are always originated from hosts and confirmed by RCMs. More precisely, the interface between a host and a RCM consists of six *request* messages from the host to the RCM with their associated *confirm* messages. In addition, *info* messages are sent to the host every time a UWB packet is received from other RCMs. Each RCM can communicate, on the UWB channel, to all the RCMs in the considered environment and each communication is associated with a message ID.

**Figure 2:**
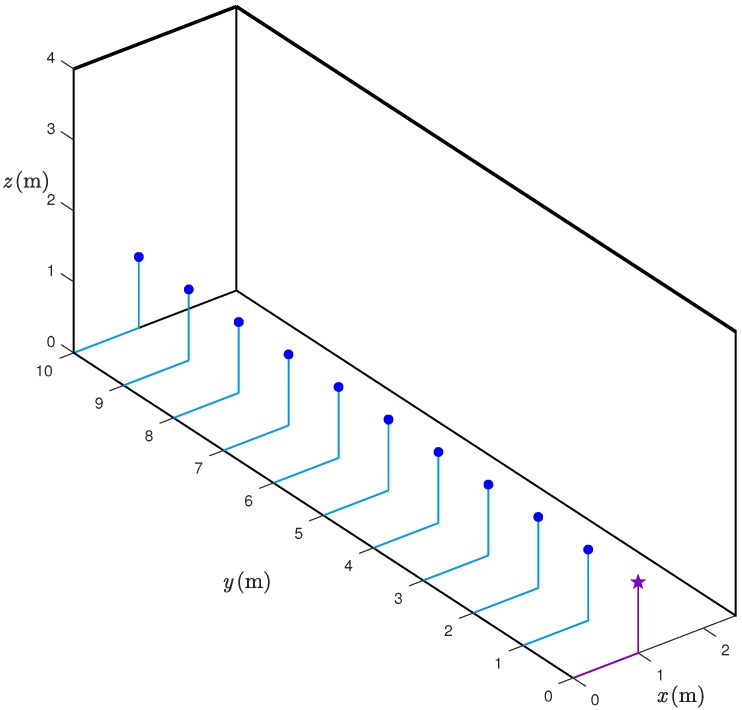
Scenario of the experimental setup: the position of the requester (purple star) and the 10 positions of the responder (blue dots) are shown.

We consider inter-sensor distances from 1 m to 10 m, as this is realistic for many indoor scenarios. The (true) distances between the sensors have been measured using a laser. The range estimates are taken at 1 m step: more precisely, for every considered distance, $N_{\mathrm{re}}=1000$ (independent) range estimates are taken—we will refer to each of the $N_{\mathrm{re}}$ estimations of the same distance as “iterations”. All the data are collected in a corridor whose width and height are 2.5 m and 4 m, respectively. The UWB sensors are placed at a height equal to 1.5 m. The considered experimental setup is shown in Figure 2. We remark that, in the setup in Figure 2, the requester and the responder are in Line-of-Sight (LoS), hence, it is reasonable to assume that the obtained results do not suffer from Non-Line-of-Sight (NLoS) propagation. The assumption of LoS between the sensors may seem strong, but is rather typical in several indoor environments. For instance, in industrial scenarios, the shelves dedicated to goods storage create corridors, where people and/or vehicles may move, and this leads to LoS communication conditions. Moreover, the approach used to derive an analytical model of the distance error can be applied also to NLoS scenarios. In the latter cases, however, the statistical model would depend on the specific scenario, e.g., on the material of possible obstacles between the nodes and on multipath components (i.e., on the geometry of the scenario). The derivation of statistical models for the range estimation error in NLoS scenarios is the object of future research.

Let us denote as ${\left\{r_{k}\right\}}_{k=1}^{10}$ the true distances between the two UWB nodes (spanning over the 10 possible responder positions, as shown in Figure 2) and as ${\left[{\hat{r}}_{k}\right]}_{j}$ the range estimate, relative to $r_{k}$, in the j-th iteration (or measurement), $j\in \{1,\ldots ,N_{\mathrm{re}}\}$, where $N_{\mathrm{re}}$ is the number of iterations. For each distance $r_{k}$, k∈{1,…,10}, and for each iteration $j\;\in \;\{\;1,\ldots ,N_{\mathrm{re}}\}$ the range estimate ${\left[{\hat{r}}_{k}\right]}_{j}$ is assumed to be modeled as follows:

(1) $${\left[{\hat{r}}_{k}\right]}_{j}≜r_{k}+{\left[{\hat{\nu }}_{k}\right]}_{j},$$

where the range error ${\left[{\hat{\nu }}_{k}\right]}_{j}$ depends on a number of factors, including the used UWB nodes and the communication scenario. The error ${\left[{\hat{\nu }}_{k}\right]}_{j}$ can be positive or negative, depending on whether the distance between the two UWB sensors is overestimated or underestimated, respectively. Since ${\left\{{\left[{\hat{\nu }}_{k}\right]}_{j}\right\}}_{j=1}^{N_{\mathrm{re}}}$ are relative to independent range measurements, it is reasonable to assume that they can be considered as independent random variables.

**Figure 3:**
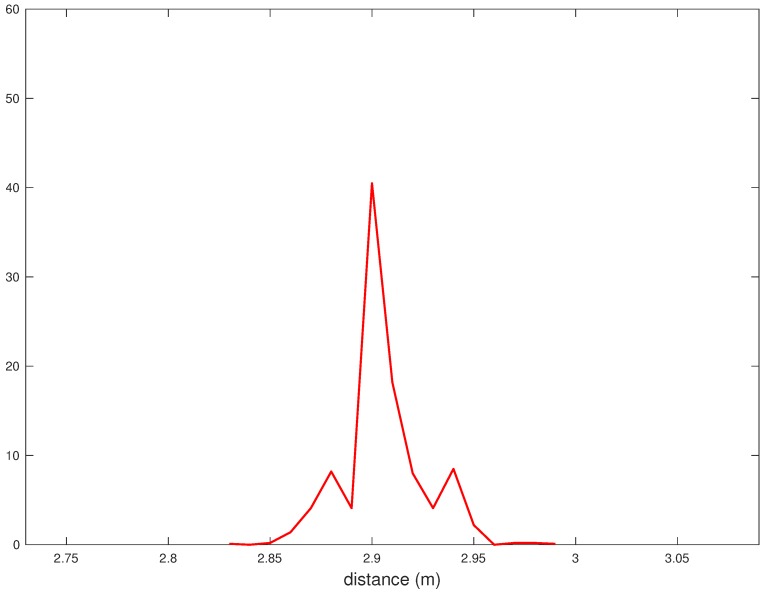
The PDF of range measurements for r=3 m.

In Figure 3, the probability Density Function (PDF) of the distance estimates $\left\{{\left[{\hat{r}}_{3}\right]}_{j}\right\}$, relative to a true distance of $r_{3}=3$ m, is shown. It can be shown that the probability mass concentrates in a 0.1 m-range around 2.9 m. In other words, there is a 0.1 m offset with respect to the true distance. The statistical model derived in the following aims at systematically correcting this offset. Let us define as ${\bar{\nu }}_{k}$ the average value of the range errors relative to the true distance $r_{k}$:

(2) $$\begin{array}{ccc} {\bar{\nu }}_{k} & ≜ & \frac{1}{N_{\mathrm{re}}}\sum_{j=1}^{N_{\mathrm{re}}}{\left[{\hat{\nu }}_{k}\right]}_{j}\;k\in \left\{1,\ldots ,10\right\}. \end{array}$$

We are now set to derive a statistical model for the range estimation error, in terms of its average value. In general, one can write:

(3) $$\hat{r}\;=\;r+\nu \left(r\right),$$

where *r* and $\hat{r}$ represent the true and estimated distances, respectively, and ν(r) is a random variable that models the range error. In particular, we assume that the range error depends on the true distance—the validity of this assumption will later be confirmed by our experimental data.

In order to characterize statistically the range estimation error in Label (3), we analyze the results obtained in the experimental scenario described above. The values of the average range errors ${\left\{{\bar{\nu }}_{k}\right\}}_{k=1}^{10}$ (evaluated according to Label (2)) are shown in the second column of Table 1 as functions of the true distances ${\left\{r_{k}\right\}}_{k=1}^{10}$.

From the results in Table 1, it can be observed that the average error ${\bar{\nu }}_{k}$ tends to increase as the distance $r_{k}$ between the two nodes increases, suggesting that the average error ${\bar{\nu }}_{k}$ could be approximated as a (linearly) increasing function of the true distance $r_{k}$.

**Figure 4:**
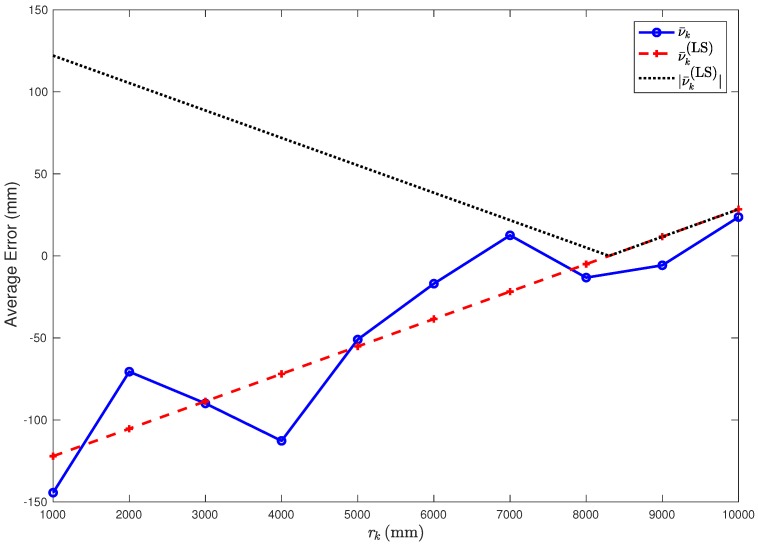
The values of the average range estimation error ${\bar{\nu }}_{k}$ (blue circles) and of its linear approximation ${\bar{\nu }}_{k}^{\left(\mathrm{LS}\right)}$ (red pluses) are shown as functions of the true distance $r_{k}$ between the two sensors.

For this reason, we consider the following Least Square (LS) approximation, based on the collected data of the range estimation error

(4) $${\bar{\nu }}_{k}≃{\bar{\nu }}_{k}^{\left(\mathrm{LS}\right)}\;=\;ar_{k}+b,$$

where ${\bar{\nu }}_{k}^{\left(\mathrm{LS}\right)}$ is the LS approximation of the average range error ${\bar{\nu }}_{k}$ and the coefficients *a* and *b* are found by applying LS estimation. More precisely, the coefficients *a* and *b* in (4) can be found by solving the linear system

(5) $$\underline{\underline{A}}\;\underline{t}\;=\;\underline{\nu },$$

where $\underline{\underline{A}}$ is a 10×2 matrix where the k-th element of its first column is $r_{k}$, k∈{1,…,10} and the elements of its second column are all equal to 1; the k-th element of $\underline{\nu }$ is ${\bar{\nu }}_{k}$ (its value depends on the collected experimental data); and $\underline{t}={\left[a,b\right]}^{T}$ is the solution vector. Applying the LS technique to solve the system of Equation (5), one obtains that the LS linear approximation of the average range estimation error ${\bar{\nu }}_{k}$ can be expressed as

(6) $${\bar{\nu }}_{k}^{\left(\mathrm{LS}\right)}\;=\;0.017r_{k}-0.138,$$

where ${\bar{\nu }}_{k}$ and $r_{k}$ are expressed in meters. The values of ${\left\{{\bar{\nu }}_{k}^{\left(\mathrm{LS}\right)}\right\}}_{k=1}^{10}$ are shown in the third column of Table 1. A graphical representation of the average error ${\bar{\nu }}_{k}$ (indicated with 95% confidence intervals), together with its linear approximation ${\bar{\nu }}_{k}^{\left(\mathrm{LS}\right)}$, is shown in Figure 4.

From Figure 4, it can be observed that the LS linear approximation fits well the experimental data. In order to better evaluate the accuracy of the LS approximation, define as $\Delta {\bar{\nu }}_{k}$ the absolute value of the difference between ${\bar{\nu }}_{k}$ and its approximation ${\bar{\nu }}_{k}^{\left(\mathrm{LS}\right)}$, i.e.,:

(7) $$\Delta {\bar{\nu }}_{k}≜\left|{\bar{\nu }}_{k}-{\bar{\nu }}_{k}^{\left(\mathrm{LS}\right)}\right|\;k\in \left\{1,\ldots ,10\right\}.$$

The values of $\Delta {\bar{\nu }}_{k}$ are shown in the fourth column of Table 1, from which it can be observed that: (i) $\Delta {\bar{\nu }}_{k}$ is, on average, shorter than 20 mm; (ii) its largest value (in correspondence to $r_{k}=4$ m) is equal to 41 mm. Hence, it can be concluded that the derived LS model for the range estimation error is accurate and, generalizing (4), one can write:

(8) $$\bar{\nu }\left(r\right)≃0.017r-0.138\;\left(m\right).$$

From (3) and (8), the estimated distance $\hat{r}$ can thus be approximated as

(9) $$\begin{array}{cc} \hat{r} & ≃1.017r-0.138\;\left(m\right). \end{array}$$

Observe that the values of $\bar{\nu }\left(r\right)$ are negative for small values of *r* (namely, $r<r_{\mathrm{th}}≃8.117$ m), while they become positive as *r* increases. In order to highlight this fact, in Figure 4, we also show the absolute values $\left\{\right|{\bar{\nu }}_{k}^{\left(\mathrm{LS}\right)}{\left|\right\}}_{k=1}^{10}$. This means that distances shorter than $r_{\mathrm{th}}$ are typically underestimated, while distances longer than $r_{\mathrm{th}}$ are overestimated. Moreover, it can be observed that the absolute value of the error ν(r) decreases as *r* increases from r=1 m to $r=r_{\mathrm{th}}$. This is due to the specific approach used by Time Domain to perform range estimates. More precisely, the range accuracy is based on the estimation of the leading edge [15]. Hence, the key to accurate range measurements is having a robust leading edge detection algorithm, which allows determining the instant at which the first energy impulse (which is likely the one associated with the direct path, in LoS conditions) arrives at the responder. In order to do so, a correlation process is performed, which involves the correlation of the received waveform with a template waveform. This process works well except when the received waveform is compressed. When transmitting at a high power, as in our scenario, then the received waveform is compressed if the responder is close to the transmitter and, therefore, the resulting correlation is also distorted, leading to a range estimation error. This problem can be addressed by reducing the transmit power or, as proposed in the current paper, by applying a proper correction.

We remark that the obtained range error estimation model is substantially similar to the one derived in [16] and used in [19], even if the indoor scenarios of the experimental distance measurement campaigns are different. The similarity between the two models, however, confirms that the proposed LS approximation of the range error is reliable (regardless of the environment and the considered set of distance measurements).

## 3. Localization: General Problem Formulation and Selected Algorithms

In this section, we consider a practical, yet general, localization problem. We remark that we do not aim at proposing new localization algorithms but at showing that the distance model derived in Section 2 is useful to improve the accuracy of (known) localization algorithms. We also remark that the results outlined in Section 2 can be considered as a calibration phase, based on the extensive experimental campaign we performed. Similar approaches can be found in [20,21].

We consider four RCMs in a bi-dimensional scenario: assuming to know the positions of three of them, we aim at finding the (unknown) position of the remaining one. We denote as Anchor Nodes (ANs) the three nodes with known positions and we denote their coordinates as

(10) $${\mathrm{AN}}_{i}≜\left[x_{i},y_{i}\right]\;i\in \left\{1,2,3\right\}.$$

Finally, we denote as Target Node (TN) the node whose position needs to be estimated and we indicate its coordinates as $\underline{u}≜\left[x,y\right]$.

In order to gather experimental results, we connect the RCM, which corresponds to the TN to a host. Proper Matlab libraries have been used for the communication between the TN and its host. First, the TN collects the range estimates from the three ANs by directly interrogating, one after another, the IDs corresponding to each of the three ANs—the TN is assumed to know the IDs of the ANs. Once the TN receives the range estimates from all the ANs, recalling that the coordinates of the ANs are known, it can compute its position by means of proper localization algorithms. In the following, two relevant localization algorithms will be considered. We remark that we do not aim at introducing new localization algorithms. Rather, our goal is to investigate the impact of the statistical characterization of the range estimation error derived in Section 2 on the accuracy of existing localization algorithms. As a matter of fact, our approach is general and can be applied to virtually any localization algorithm.

In order to statistically characterize the accuracy of the position estimates, for each localization problem, we consider 1000 location estimations (denoted as iterations). In the following, we denote: ${\left[{\hat{r}}_{i}\right]}_{j}$ as the estimated distance between ${\mathrm{AN}}_{i}$, i∈{1,2,3}, and the TN in the j-th iteration; and ${\left[\underline{\hat{u}}\right]}_{j}≜\left[{\left[\hat{x}\right]}_{j},{\left[\hat{y}\right]}_{j}\right]$ as the estimated TN coordinates in the j-th iteration, j∈{1,…,1000}.

The two considered localization algorithms are described in Section 3.1 and Section 3.2, respectively.

### 3.1. Circumference Intersection (CI) Algorithm

In this subsection, a simple localization algorithm, denoted as Circumference Intersection (CI), is presented. A similar approach is introduced in [22]. In order to better explain the CI algorithm, let us make a few geometrical considerations on the localization problem. The knowledge of the coordinates of ${\mathrm{AN}}_{i}$ and of the true distances ${\left\{r_{i}\right\}}_{i=1}^{3}$ would allow obtaining the true position of the TN. As a matter of fact, the TN has to lay on each of the circumferences ${\left\{C_{i}\right\}}_{i=1}^{3}$, centered in ${\mathrm{AN}}_{i}$ and with radii ${\left\{r_{i}\right\}}_{i=1}^{3}$ identified by the following equations:

(11) $$\left\{\begin{array}{c} {\left(x-x_{1}\right)}^{2}+{\left(y-y_{1}\right)}^{2}=r_{1}^{2},\\ {\left(x-x_{2}\right)}^{2}+{\left(y-y_{2}\right)}^{2}=r_{2}^{2},\\ {\left(x-x_{3}\right)}^{2}+{\left(y-y_{3}\right)}^{2}=r_{3}^{2}. \end{array}\right.$$

Hence, the TN coordinates $\underline{u}\;=\;\left[x,y\right]$ could be found as the (unique) point where the three circumferences ${\left\{C_{i}\right\}}_{i=1}^{3}$ intersect.

Since the values of the true distances ${\left\{r_{i}\right\}}_{i=1}^{3}$ are unknown, one can only rely on their estimates ${\left[{\hat{r}}_{i}\right]}_{j}$, j∈{1…,1000}, i∈{1,2,3}. For this reason, at each step j∈{1,…,1000}, the system (11) should be replaced by the following:

(12) $$\left\{\begin{array}{c} {\left({\left[x\right]}_{j}-x_{1}\right)}^{2}+{\left({\left[y\right]}_{j}-y_{1}\right)}^{2}={\left[{\left[{\hat{r}}_{1}\right]}_{j}\right]}^{2},\\ {\left({\left[x\right]}_{j}-x_{2}\right)}^{2}+{\left({\left[y\right]}_{j}-y_{2}\right)}^{2}={\left[{\left[{\hat{r}}_{2}\right]}_{j}\right]}^{2},\\ {\left({\left[x\right]}_{j}-x_{3}\right)}^{2}+{\left({\left[y\right]}_{j}-y_{3}\right)}^{2}={\left[{\left[{\hat{r}}_{3}\right]}_{j}\right]}^{2}. \end{array}\right.$$

The equations in (12) refer to the three circumferences, denoted as ${\left\{{\left[{\hat{C}}_{i}\right]}_{j}\right\}}_{i=1}^{3}$, centered in ${\mathrm{AN}}_{i}$ (as in (11)) with radii ${\left\{{\left[{\hat{r}}_{i}\right]}_{j}\right\}}_{i=1}^{3}$. While the three circumferences ${\left\{C_{i}\right\}}_{i=1}^{3}$ intersect in a unique point (which corresponds to the true TN position), due to the errors that affect the range estimates ${\left\{{\left[{\hat{r}}_{i}\right]}_{j}\right\}}_{i=1}^{3}$, the three circumferences ${\left\{{\left[{\hat{C}}_{i}\right]}_{j}\right\}}_{i=1}^{3}$ may not intersect in a unique point.

In order to find the TN position estimates in the j-th iteration, denoted as ${\left[\underline{\hat{u}}\right]}_{j}\;=\;\left[{\left[\hat{x}\right]}_{j},{\left[\hat{y}\right]}_{j}\right]$, it is therefore necessary to consider a proper localization approach: rather than looking for the intersection of the three circumferences identified by (12) (which would not lead to any solution), we intersect pairs of them (namely, 32=3 pairs). More precisely, for each iteration j∈{1,…,1000}, let us define the following three sets (each of which contains two points), obtained by intersecting the three different pairs of circumferences:

(13) $$\begin{array}{ccc} \left\{P_{12},Q_{12}\right\} & ≜ & {\left[{\hat{C}}_{1}\right]}_{j}\cap {\left[{\hat{C}}_{2}\right]}_{j}, \end{array}$$

(14) $$\begin{array}{ccc} \left\{P_{13},Q_{13}\right\} & ≜ & {\left[{\hat{C}}_{1}\right]}_{j}\cap {\left[{\hat{C}}_{3}\right]}_{j}, \end{array}$$

(15) $$\begin{array}{ccc} \left\{P_{23},Q_{23}\right\} & ≜ & {\left[{\hat{C}}_{2}\right]}_{j}\cap {\left[{\hat{C}}_{3}\right]}_{j}. \end{array}$$

We then choose a point from each of the three sets, in such a way that the three selected points are the nearest ones to each other. Finally, the TN position estimate is found as the center of gravity of these three points.

We remark that the intersection between two circumferences could also be an empty set. In this case, the localization algorithm needs to be slightly modified. Without loss of generality, let us assume that ${\left[{\hat{C}}_{1}\right]}_{j}$ and ${\left[{\hat{C}}_{2}\right]}_{j}$ do not intersect. We can then find $P_{12}\in {\left[{\hat{C}}_{1}\right]}_{j}$ and $Q_{12}\in {\left[{\hat{C}}_{2}\right]}_{j}$ as the two points of the two circumferences, which are nearest to each other, namely:

(16) $$\left\{P_{12},Q_{12}\right\}\;=\;{\mathrm{argmin}}_{P\in {\left[{\hat{C}}_{1}\right]}_{j},Q\in {\left[{\hat{C}}_{2}\right]}_{j}}||P-Q||.$$

At this point, if the distance $\left|\right|P_{12}-Q_{12}\left|\right|$ between $P_{12}$ and $Q_{12}$ is lower than a given threshold, (In the following, we will consider a threshold equal to 500 mm.), then the two points are considered as if they were the two points of intersection between ${\left[{\hat{C}}_{1}\right]}_{j}$ and ${\left[{\hat{C}}_{2}\right]}_{j}$. Otherwise, they are ignored and the localization strategy is only based on the remaining pairs of circumferences. A similar strategy can be carried out in the case that more than one pair of circumferences does not intersect.

### 3.2. Two-Stage Maximum-Likelihood (TSML) Algorithm

In the literature, this algorithm is considered as optimal as it allows attaining the Cramer–Rao bound, which is a universal lower bound for the variance of an estimator [17]. For each iteration j∈{1,…,1000}, the starting point of this algorithm is the system given by (12), which is a quadratic system in the two unknowns *x* and *y*. To solve system (12), we consider a two-step approach, based on a Maximum-Likelihood (ML) technique, which has been proposed in [17] and is known as the Two-Stage Maximum-Likelihood Time-Of-Arrival (TSML-TOA) algorithm (for short, TSML).

The first phase of the TSML algorithm relies on introducing the variable

(17) $$n≜\left|\right|\underline{u}{\left|\right|}^{2}\;=\;x^{2}+y^{2}$$

so that system (12) can be rewritten, in matrix notation, as

(18) $$\underline{\underline{G}}\;{\underline{\hat{\omega }}}_{1}=\hat{\underline{h}},$$

where:

(19) $$\begin{array}{c} \underline{\underline{G}}=-2\left(\begin{array}{ccc} x_{1}\; & \;y_{1}\; & \;-0.5\\ x_{2}\; & \;y_{2}\; & \;-0.5\\ x_{3}\; & \;y_{3}\; & \;-0.5 \end{array}\right)\;{\underline{\hat{\omega }}}_{1}=\left(\begin{array}{c} x\\ y\\ n \end{array}\right)\;\hat{\underline{h}}=\left(\begin{array}{c} {\left[{\left[{\hat{r}}_{1}\right]}_{j}\right]}^{2}-K_{1}\\ {\left[{\left[{\hat{r}}_{2}\right]}_{j}\right]}^{2}-K_{2}\\ {\left[{\left[{\hat{r}}_{3}\right]}_{j}\right]}^{2}-K_{3} \end{array}\right) \end{array}$$

and $K_{i}≜x_{i}^{2}+y_{i}^{2}$, ∀i∈{1,2,3}. While (18) might look like a linear system, in reality, it is not, since, according to (17), the third element of the solution vector ${\underline{\hat{\omega }}}_{1}$ depends on the first two. The solution ${\underline{\hat{\omega }}}_{1}$ of the system (18) can be determined through a ML approach. The second stage of the algorithm is meant to take into account the dependence of *n* on the other unknowns of (18) and involves the solution of the following system:

(20) $${\underline{\underline{G}}}^{\prime }\;{\underline{\hat{\omega }}}_{2}={\underline{\hat{h}}}^{\prime },$$

where

$$\begin{array}{c} {\underline{\underline{G}}}^{\prime }=\left(\begin{array}{cc} 1\; & \;0\\ 0\; & \;1\\ 1\; & \;1 \end{array}\right)\;{\underline{\hat{\omega }}}_{2}=\left(\begin{array}{c} x^{2}\\ y^{2} \end{array}\right)\;{\underline{\hat{h}}}^{\prime }\;=\;\left(\begin{array}{c} {\left[{\underline{\hat{\omega }}}_{1}\right]}_{1}^{2}\\ {\left[{\underline{\hat{\omega }}}_{1}\right]}_{2}^{2}\\ {\left[{\underline{\hat{\omega }}}_{1}\right]}_{3} \end{array}\right) \end{array}$$

and ${\left[{\underline{\hat{\omega }}}_{1}\right]}_{j}$ denotes the j-th component of ${\underline{\hat{\omega }}}_{1}$. System (20) can be solved, once again, through the ML technique—the interested reader is referred to [17] for further details. Finally, the position estimate can be expressed as:

$$\underline{u}=\underline{\underline{U}}{\left[\sqrt{{\left[{\underline{\hat{w}}}_{2}\right]}_{1}},\sqrt{{\left[{\underline{\hat{w}}}_{2}\right]}_{2}}\right]}^{T},$$

where $\underline{\underline{U}}=\mathrm{diag}\left(\mathrm{sign}\left({\underline{\hat{\omega }}}_{1}\right)\right)$ [17].

## 4. Localization: Experimental Performance Investigation

In this section, the statistical model for the range estimation error derived in Section 2 is applied to practical localization problems, in order to analyze its impact on the accuracy of the obtained position estimate. In particular, we show how the statistical model derived in Section 2 is effective in improving (with gains up to 66%) the performance of the localization algorithms (CI and TSML) recalled in Section 3.

From (9), it follows that:

(21) $$r≃\frac{\hat{r}+0.138}{1.017}.$$

Instead of applying the localization strategies described in Section 3.1 and Section 3.2 with the distances ${\left\{{\left[{\hat{r}}_{i}\right]}_{j}\right\}}_{i=1}^{3}$ (estimated from the nodes), we consider the following modified distances, obtained by correcting ${\left\{{\left[{\hat{r}}_{i}\right]}_{j}\right\}}_{i=1}^{3}$ according to (21):

(22) $$\;bf{\left[{\overset{ˇ}{r}}_{i}\right]}_{j}≜\frac{{\left[{\hat{r}}_{i}\right]}_{j}+0.138}{1.017}\;i\in \left\{1,2,3\right\}\;\;\;\;j\in \left\{1,\ldots ,1000\right\}.\;$$

In particular, denote: as ${\left[\hat{d}\right]}_{j}$ the distance between the true TN position and its estimate in the j-th iteration, *without* taking into account the statistical model of the range estimation error; and, as ${\left[\overset{ˇ}{d}\right]}_{j},$ the distance between the true TN position and its estimate in the j-th iteration *with* the use of the proposed statistical model, i.e., (22). Therefore, one can write:

(23) $$\begin{array}{c} {\left[\hat{d}\right]}_{j}≜\left|\right|\underline{u}-{\left[\underline{\hat{u}}\right]}_{j}||\;{\left[\overset{ˇ}{d}\right]}_{j}≜\left|\right|\underline{u}-{\left[\underline{\overset{ˇ}{u}}\right]}_{j}\left|\right|, \end{array}$$

where ${\left[\underline{\overset{ˇ}{u}}\right]}_{j}≜\left[{\left[\overset{ˇ}{x}\right]}_{j},{\left[\overset{ˇ}{y}\right]}_{j}\right]$ are the estimated coordinates of the TN in the j-th iteration with the modified distances given by (22). Moreover, define the average values (averaged over the considered 1000 iterations) of the distances between true and estimated TN position defined in (23), namely:

(24) $$\begin{array}{cc} {\hat{d}}_{\mathrm{avg}} & ≜\frac{1}{1000}\sum_{j=1}^{1000}{\left[\hat{d}\right]}_{j},\\ {\overset{ˇ}{d}}_{\mathrm{avg}} & ≜\frac{1}{1000}\sum_{j=1}^{1000}{\left[\overset{ˇ}{d}\right]}_{j}. \end{array}$$

Finally, we define also the maximum values of the distances between true and estimated TN positions, namely:

(25) $$\begin{array}{cc} {\hat{d}}_{\max} & ≜\max_{j\in \{1,\ldots ,1000\}}{\left[\hat{d}\right]}_{j},\\ {\overset{ˇ}{d}}_{\max} & ≜\max_{j\in \{1,\ldots ,1000\}}{\left[\overset{ˇ}{d}\right]}_{j}. \end{array}$$

**Figure 5:**
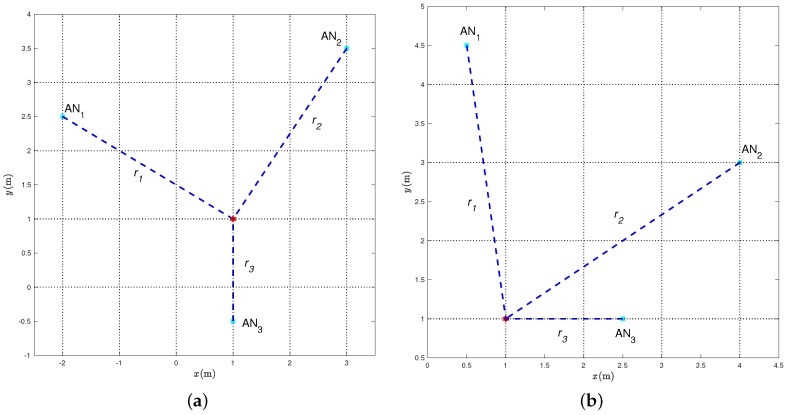
Considered geometrical scenarios: (**a**) “good” and (**b**) “bad.” In both cases, the true position of the TN (red star) is shown, together with the positions of three ANs (blue stars). The distances ${\left\{r_{i}\right\}}_{i=1}^{3}$ are also shown.

The following experimental results are relative to the two relevant scenarios shown in Figure 5a and Figure 5b, which are representative of “good” and “bad” topological conditions, respectively. In both cases, the range estimates have been obtained in a square room with side 5 m, namely in a different environment with respect to that where the previously described measurement campaign was performed. The main difference between the two scenarios in Figure 5 is that, in the first case, the TN lies inside the convex hull of the three ANs, while, in the latter, the TN lies outside the convex hull of the three ANs. We remark that the statistical model of the range estimation error was derived in Section 2 through an experimental campaign in a corridor (see Figure 2). Its applicability to another scenario (a square room) is a further indicator of its validity and robustness.

We then apply both the CI and the TSML algorithms, described in Section 3.1 and Section 3.2, respectively, to both scenarios in Figure 5.

### 4.1. “Good” Geometrical Scenario

First, we consider the scenario shown in Figure 5a, where the coordinates of the ANs, expressed in meters, are

(26) $$\begin{array}{c} {\mathrm{AN}}_{1}=\left[-2,2.5\right]\;{\mathrm{AN}}_{2}=\left[3,3.5\right]\;{\mathrm{AN}}_{3}=\left[1,-0.5\right] \end{array}$$

while the TN coordinates, expressed in meters, are

(27) $$\begin{array}{c} \underline{u}=\left[1,1\right]. \end{array}$$

The (true) distances ${\left\{r_{i}\right\}}_{i=1}^{3}$ between each AN and the TN are also shown. From (26) and (27), it follows that their values are:

(28) $$\begin{array}{c} r_{1}=\frac{3}{2}\sqrt{5}\;m\;r_{2}=\frac{\sqrt{41}}{2}\;m\;r_{3}=1.5\;m. \end{array}$$

**Figure 6:**
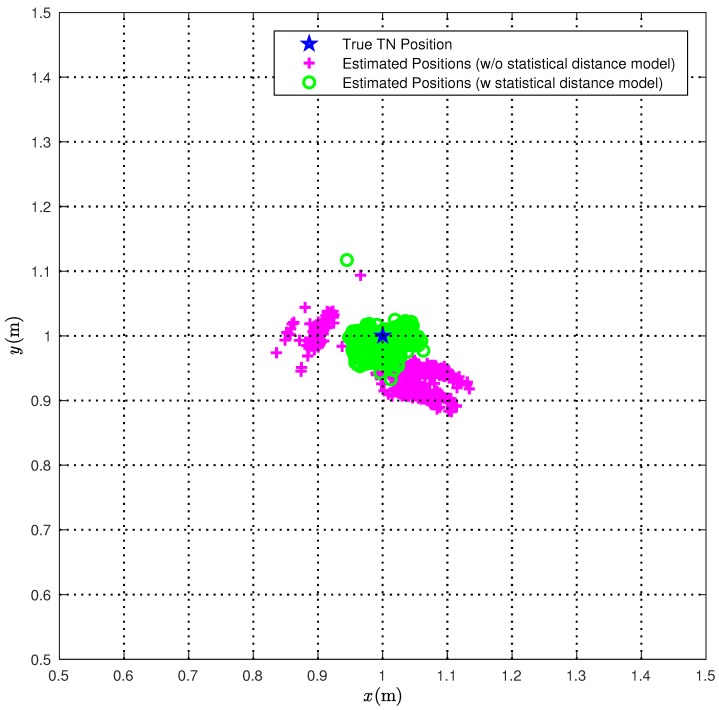
The 1000 position estimates obtained with the CI localization algorithm are shown (magenta plus), together with the 1000 position estimates obtained when taking into account the statistical model for range estimates (green circles) and the TN (blue star).

We first consider the CI algorithm. In Figure 6, we compare the true TN position (red star) with the position estimates obtained over 1000 measurements with (green circles) or without (magenta pluses) the correction (22).

**Figure 7:**
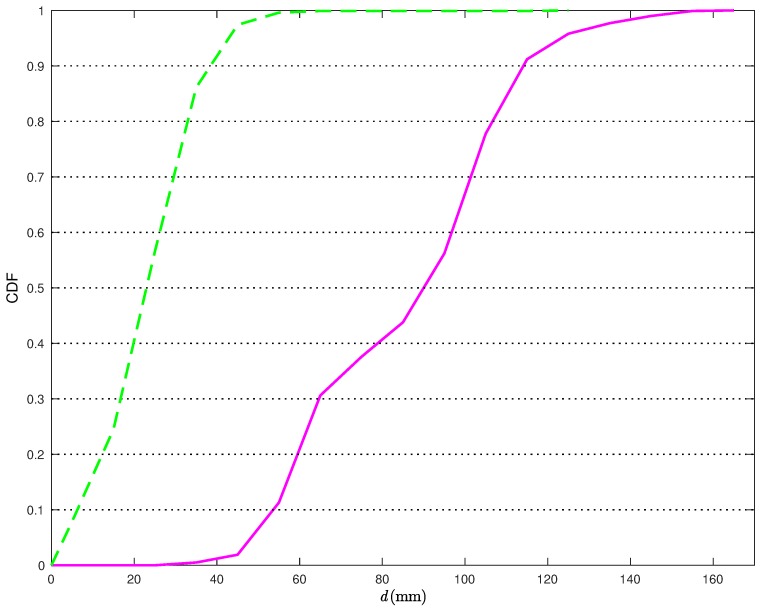
CDFs of the distance errors obtained with the CI localization algorithm in Figure 6 without (solid line) and with (dashed line) the application of the statistical model on the distance estimation error.

From Figure 6, it is apparent that the proposed statistical model allows for improving the performance accuracy of the CI localization algorithm. In Figure 7, the Cumulative Distribution Functions (CDFs) of the distances between the true TN position and its estimates are shown.

More precisely, the solid line is relative to the position estimates obtained without the statistical model for the distance estimates, while the dashed line is relative to the position estimates obtained applying the corrections in (22). From Figure 7, it can be observed that, when applying the proposed correction, the distance error is smaller than 40 mm about 90% of the times. At the opposite, if the statistical model for the distance estimates is not used, the distance error is smaller than 40 mm less than 2% of the times. Considering a probability equal to 0.9, it can be concluded that the average distance error reduces from 117 mm to 38 mm, with a relative performance improvement (in terms of reduction of the average distance error) around 66%.

In Table 2, we show the average and the maximum distances between the true TN position and its estimates obtained with and without considering the statistical model for the range estimation error. From the second column of Table 2, it can be observed that using the values of the distances ${\left\{{\overset{ˇ}{r}}_{i}\right\}}_{i=1}^{3}$, obtained with the correction (22), instead of the values of the distances ${\left\{{\hat{r}}_{i}\right\}}_{i=1}^{3}$, directly estimated by the nodes, allows for reducing the average range estimation error. As a matter of fact, the value of ${\overset{ˇ}{d}}_{\mathrm{avg}}$ is 60 mm lower than ${\hat{d}}_{\mathrm{avg}}$ (corresponding to a reduction of 68%). Similarly, the maximum range error ${\overset{ˇ}{d}}_{\max}$, obtained using ${\left\{{\overset{ˇ}{r}}_{i}\right\}}_{i=1}^{3}$, is 40 mm shorter (corresponding to a reduction of 22%) than the maximum range error ${\hat{d}}_{\max}$ obtained using ${\left\{{\hat{r}}_{i}\right\}}_{i=1}^{3}$.

**Figure 8:**
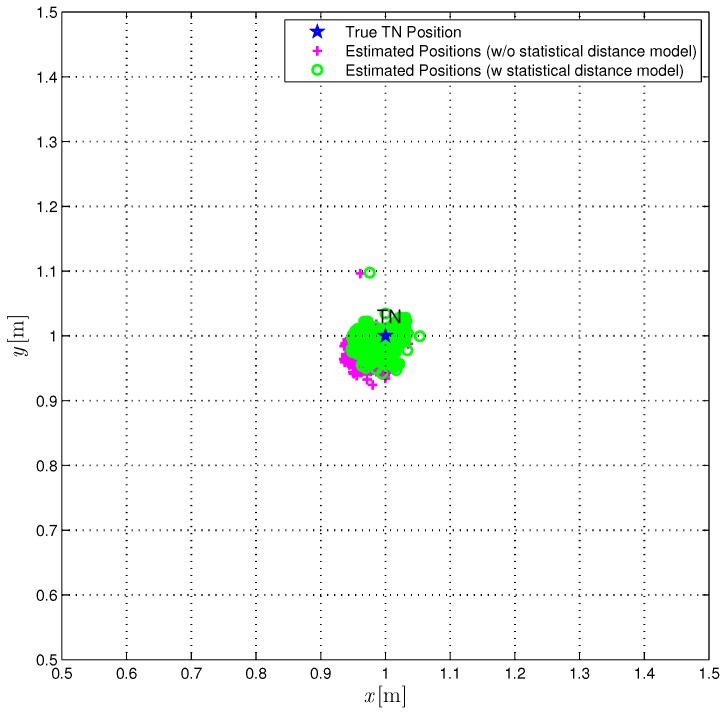
The 1000 position estimates obtained with the TSML localization algorithm are shown (magenta plus), together with the 1000 position estimates obtained when taking into account the statistical model for range estimates (green circles) and the TN (blue star).

**Figure 9:**
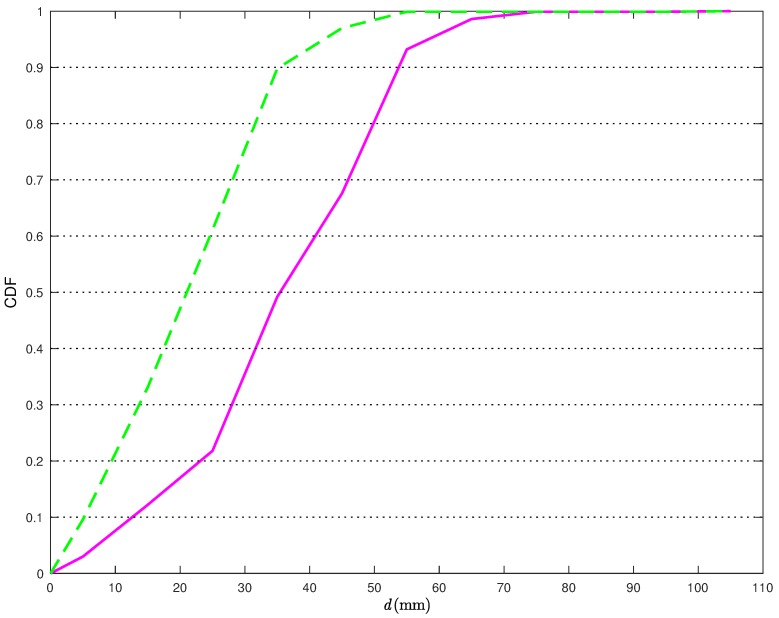
CDFs of the distance errors obtained with the TSML localization algorithm in Figure 8 without (solid line) and with (dashed line) the statistical model.

The true TN position (red star) is compared with the position estimates obtained over 1000 iterations with (green circles) or without (magenta pluses) the correction (22). From Figure 8, it can be observed that the use of the proposed statistical model improves the performance accuracy of the TSML localization algorithm as well, even if in this case the improvement is limited. In Figure 9, the CDFs of the distances between the true TN position and its estimates (obtained according to the TSML algorithm) are shown. In Figure 9, the performance of the TSML localization algorithm is investigated. More precisely, the solid line is relative to the position estimates obtained without the statistical model for the distance estimates, while the dashed line is relative to the position estimates obtained using the corrections (22). Unlike Figure 7, the two CDFs are similar and the distance error turns out to be smaller than 60 mm, more than 95% of the times even without correction. Considering a probability equal to 0.9, the relative reduction of the average distance estimation error, using the proposed statical model, is around 33%. As a matter of fact, from the third column of Table 2, it can be observed that the value of ${\overset{ˇ}{d}}_{\mathrm{avg}}$ is only 14 mm smaller (corresponding to a reduction of 35%) than ${\hat{d}}_{\mathrm{avg}}$, while the value of ${\overset{ˇ}{d}}_{\max}$ is only 3 mm (corresponding to a reduction of 3%) shorter than ${\hat{d}}_{\max}$.

### 4.2. “Bad” Geometrical Scenario

We now consider the scenario shown in Figure 5b, where the TN is outside the convex hull of the ANs, which have the following coordinates (in meters):

(29) $$\begin{array}{c} {\mathrm{AN}}_{1}=\left[0.5,4.5\right]\;{\mathrm{AN}}_{2}=\left[4,3\right]\;{\mathrm{AN}}_{3}=\left[2.5,1\right], \end{array}$$

while the position of the TN is the same as in Section 4.1, namely:

(30) $$\begin{array}{c} \underline{u}=\left[1,1\right]. \end{array}$$

From (27) and (29), it follows that the values of the distances ${\left\{r_{i}\right\}}_{i=1}^{3}$ are:

(31) $$\begin{array}{c} r_{1}=\frac{5}{2}\sqrt{2}\;m\;r_{2}=\sqrt{13}\;m\;r_{3}=1.5\;m. \end{array}$$

As for the previous (“good”) scenario, we localize the TN by applying the CI and the TSML algorithms.

**Figure 10:**
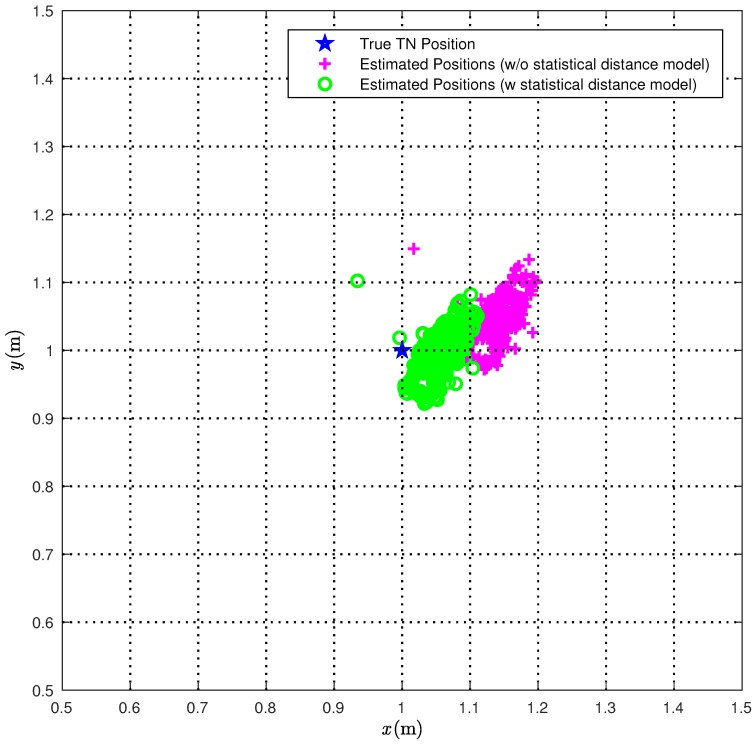
The 1000 position estimates obtained with the CI localization algorithm are shown (magenta plus), together with the 1000 position estimates obtained when taking into account the statistical model for range estimates (green circles) and the TN (blue star).

In Figure 10, we directly compare the true TN position (red star) with the position estimates obtained according to the CI algorithm over 1000 iterations with (green circles) or without (magenta pluses) the correction (22). The obtained results show that the use of the proposed statistical model improves significantly the performance accuracy of the CI localization algorithm.

**Figure 11:**
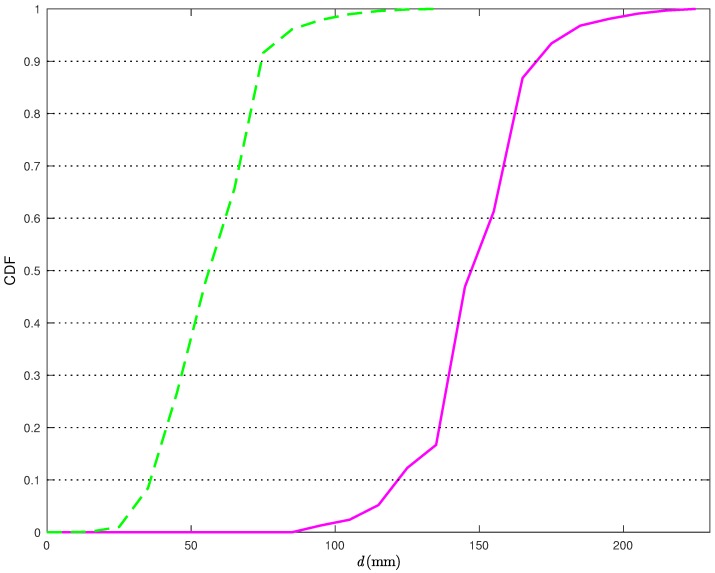
CDFs of the distance errors obtained with the CI localization algorithm in Figure 10 without (solid line) and with (dashed line) the statistical model.

In Figure 11, the CDFs of the distances between the true TN position and its estimates in the scenario of Figure 5b are shown without (solid line) and with (dashed line) the correction (22).

From Figure 11, it can be observed that, when applying the statistical model, the distance error is smaller than 90 mm more than 95% of the times. At the opposite, if the statistical model for the distance estimates is not used, the distance error is smaller than 90 mm only in 2% of the times. Moreover, in the latter case, the probability of having distance errors smaller than a given threshold is above 95% only if one can tolerate errors up to 190 mm. Considering a probability equal to 0.9, the relative reduction of the average distance estimation error, using the proposed statical model, is around 56%.

In Table 3, we show the average and the maximum distances between the true TN position and its estimates obtained with and without considering the statistical model for the range estimation error. In particular, from the second column of Table 3, it can be observed that the value of ${\overset{ˇ}{d}}_{\mathrm{avg}}$ obtained using the distances ${\left\{{\overset{ˇ}{r}}_{i}\right\}}_{i=1}^{3}$ is 90 mm shorter (corresponding to a reduction of 59%) than the value of ${\hat{d}}_{\mathrm{avg}}$, obtained by applying the CI localization algorithm with the distances ${\left\{{\hat{r}}_{i}\right\}}_{i=1}^{3}$ directly estimated by the nodes. Similarly, the maximum range error ${\overset{ˇ}{d}}_{\max}$ obtained using ${\left\{{\overset{ˇ}{r}}_{i}\right\}}_{i=1}^{3}$ is reduced by 100 mm (corresponding to a reduction of 43%) with respect to the value of ${\hat{d}}_{\max}$ obtained using ${\left\{{\hat{r}}_{i}\right\}}_{i=1}^{3}$.

**Figure 12:**
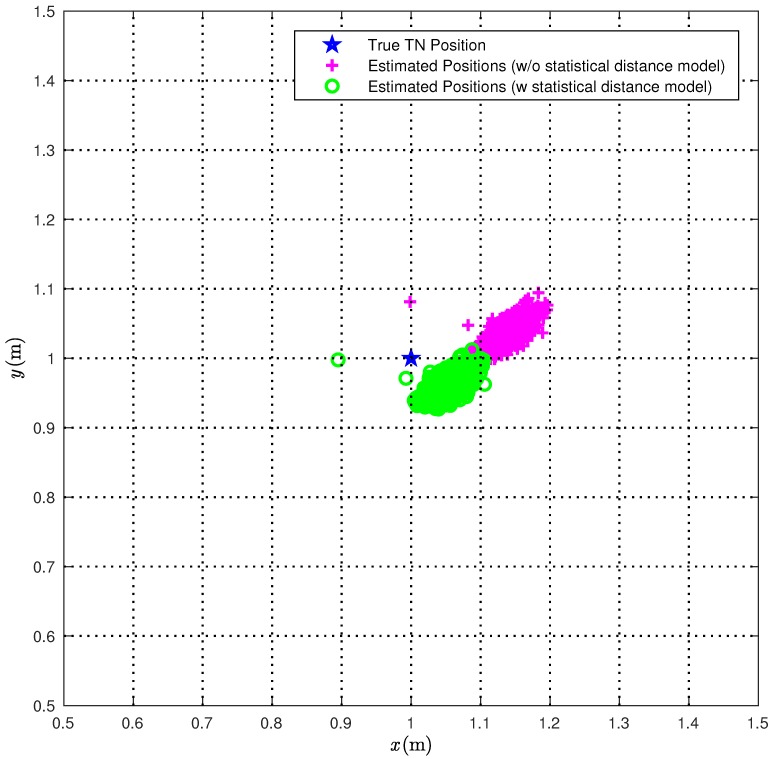
The 1000 position estimates obtained with the TSML localization algorithm are shown (magenta plus), together with the 1000 position estimates obtained when taking into account the statistical model for range estimates (green circles) and the TN (blue star).

**Figure 13:**
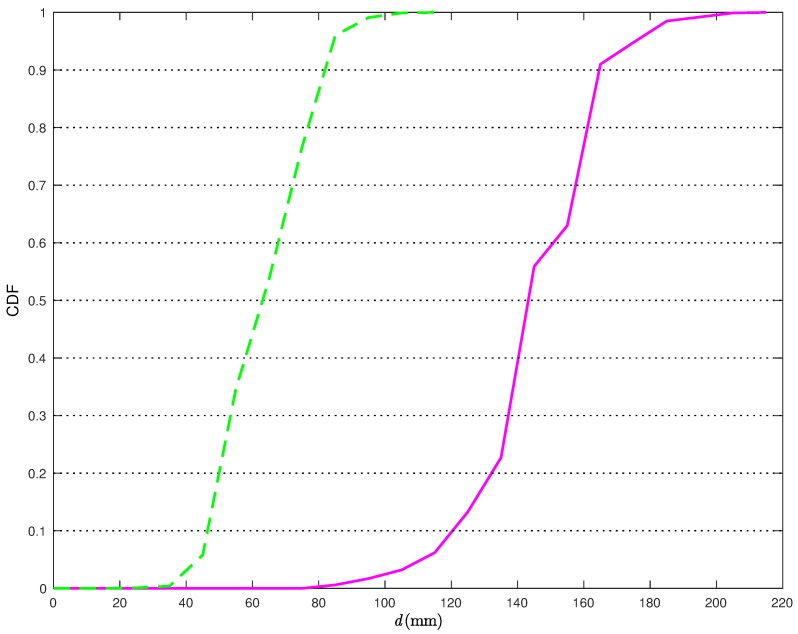
CDFs of the distance errors obtained with the TSML localization algorithm in Figure 12 without (solid line) and with (dashed line) the statistical model.

Finally, Figure 12 refers to the TSML localization algorithm. The true TN position (red star) is compared with the position estimates obtained over 1000 iterations with (green circles) or without (magenta pluses) correction (22). From Figure 12, it can be observed that the use of the proposed statistical model is beneficial also for the TSML localization algorithm, and that the improvement is comparable with the one of the CI algorithm. In Figure 13, the CDFs of the distances between the true TN position and its estimates, without (solid line) and with (dashed line) the correction (22), are shown.

Once more, the results in Figure 13 show that the use of the correction (22) makes the distance error smaller than 90 mm more than 95% of the times. At the opposite, if the statistical model for the distance estimates is not used, the distance error is rarely smaller than 90 mm, as when using the CI localization algorithm (Figure 11), the probability of having distance errors lower than a given threshold is above 90% only if one can tolerate errors up to 190 mm. As a matter of fact, from the third column of Table 3, it can be observed that the value of ${\overset{ˇ}{d}}_{\mathrm{avg}}$ is nearly 90 mm shorter (corresponding to a reduction of 54%) than ${\hat{d}}_{\mathrm{avg}}$, while the value of ${\overset{ˇ}{d}}_{\max}$ is nearly 100 mm smaller (corresponding to a reduction of 46%) than ${\hat{d}}_{\max}$, as in the case with the CI algorithm. Considering a probability equal to 0.9 in Figure 13, the relative reduction of the average distance estimation error, using the proposed statical model, is around 50%.

### 4.3. Discussion

The proposed “range error reduction” approach is based on two phases: (i) in the first (“calibration") phase, a range estimation error model is derived, on the basis of experimental measurements; (ii) in a second phase, the derived model is used in practical localization algorithms, by simply correcting the estimated pairwise distances before using them in the localization algorithms. The outcome of the first phase depends on the used nodes (in our case, the Time Domain RCMs), but the approach is general.

In Table 4, we summarize the obtained results in terms of algorithms’ sensitivity to error. In particular, considering a CDF value equal to 0.9, we extract the corresponding distances. In other words, we identify the limiting value within which the distance error lays with probability 0.9. In order to do so, we define ${{\left[\hat{d}\right]}_{j}}^{\mathrm{th}}$ as the value, which satisfies

(32) $$P({\left[\hat{d}\right]}_{j}<{{\left[\hat{d}\right]}_{j}}^{\mathrm{th}})=0.9,$$

and we define ${{\left[\overset{ˇ}{d}\right]}_{j}}^{\mathrm{th}}$ as the value that satisfies

(33) $$P({\left[\overset{ˇ}{d}\right]}_{j}<{{\left[\overset{ˇ}{d}\right]}_{j}}^{\mathrm{th}})=0.9$$

Moreover, we define the difference between these two values as $\Delta ={{\left[\hat{d}\right]}_{j}}^{\mathrm{th}}-{{\left[\overset{ˇ}{d}\right]}_{j}}^{\mathrm{th}}$. From Table 4, it can be observed that the CI algorithm particularly benefits from the range estimates correction proposed by our model. As a matter of fact, when considering the “good” scenario, the value of Δ relative to the CI algorithm is 79 mm and it is larger than that relative to the TSML algorithm, which is 19 mm. Also in the “bad” scenario, the CI algorithm takes advantage of the distance correction more than the TSML algorithm, even though the difference between the two values of Δ is less significant (namely, 11 mm). This is probably due by the fact that the TSML algorithm is known to be more robust than the CI algorithm [14]. Comparing the results in Table 2 with those in Table 3, it can be observed, as expected, that both the considered localization algorithms (namely, CI and TSML) guarantee a better accuracy in the scenario where the TN is inside the convex hull of the ANs. Moreover, the performance improvement due to the use of the distances ${\left\{{\overset{ˇ}{r}}_{i}\right\}}_{i=1}^{3}$, obtained according to (21), is more evident if the TN is outside the convex hull of the ANs, i.e., with a “worse” geometric configuration of the nodes. In fact, when the target is outside the convex hull, then the “diversity” of the anchors reduces and the performance of the algorithms degrades. This is intuitively evident with the CI algorithm, as the intersecting circumferences are much more affected by measurement noise. It can thus be concluded that the geometry of the scenario has an impact on the benefits brought by the use of the proposed statistical model of the range estimation error. More precisely, when the geometry is “favorable”, i.e., when the TN is inside the convex hull of ${\left\{{\mathrm{AN}}_{i}\right\}}_{i=1}^{3}$, the use of the corrected (according to (21)) distances ${\left\{{\overset{ˇ}{r}}_{i}\right\}}_{i=1}^{3}$ is relevant with a “weak” localization algorithm (e.g., the CI algorithm), while it becomes limited with a “strong” localization algorithm (e.g., the TSML algorithm). On the contrary, with an “unfavorable” geometry, i.e., the TN is outside the convex hull of ${\left\{{\mathrm{AN}}_{i}\right\}}_{i=1}^{3}$, the use of the corrected distances ${\left\{{\overset{ˇ}{r}}_{i}\right\}}_{i=1}^{3}$ significantly improves the performance also with a “strong” localization (namely, the TSML) algorithm. This suggests that the proposed approach is attractive in practical scenarios (e.g., industrial scenarios), where the position of the TN may vary significantly, moving often outside the convex hull of fixed ANs.

## 5. Conclusions

In this paper, we have first carried out, using Time Domain RCMs, an extensive experimental campaign of range measurements for various pairwise distances (between 1 m and 10 m). The average range estimation error has been statistically analyzed and the obtained results show that it is well approximated as a linear function of the true distance between the considered pair of nodes. Then, the derived statistical model has been applied to two different localization algorithms (namely, CI and TSML) in two realistic localization scenarios (with different nodes’ topology) in a different indoor environment. More precisely, the proposed model is used to correct the estimated pairwise distances before feeding the localization algorithms. We remark that the proposed model is valid in LoS scenarios, but the same ideas could be generalized to take into account NLoS scenarios, in which the statistical model would depend on the specific geometry of the environment and on the material of obstacles between the considered nodes. Our results show that the use of the proposed model allows for improving the performance of the considered localization algorithms. In particular, the more the improvement is pronounced, the less favorable is the nodes’ geometry. This makes the proposed approach very attractive in scenarios where the geometry of the localization problem may vary significantly, e.g., in industrial scenarios where a node (to be localized) may move among fixed (anchor) nodes.

## Figures and Tables

**Table 1:** This table shows the values of ${\bar{\nu }}_{k}$ (second column), their linear approximation ${\bar{\nu }}_{k}^{\left(LS\right)}$ (third column), and the absolute value $\Delta {\bar{\nu }}_{k}$ of their difference (fourth column), as a function of the true distances $r_{k}$.

$r_{k}$ (m)	${\bar{\nu }}_{k}$ (mm)	${\bar{\nu }}_{k}^{\left(\mathrm{LS}\right)}$ (mm)	$\Delta {\bar{\nu }}_{k}$ (mm)
1	−144	−122	22
2	−70	−105	35
3	−89	−89	0
4	−112	−72	40
5	−51	−55	4
6	−17	−38	21
7	12	−22	34
8	−13	−5	8
9	−6	12	18
10	24	28	4

**Table 2:** The average and the maximum distance between the true TN position and its estimates with (${\overset{ˇ}{d}}_{\mathrm{avg}}$, ${\overset{ˇ}{d}}_{\max}$) and without (${\hat{d}}_{\mathrm{avg}}$, ${\hat{d}}_{\max}$) the use of the statistical model, when using the CI algorithm and the TSML algorithm, are shown.

Scenario 1	CI Algorithm	TSML Algorithm
${\hat{d}}_{\mathrm{avg}}$ (mm)	90	40
${\overset{ˇ}{d}}_{\mathrm{avg}}$ (mm)	28	26
${\hat{d}}_{\max}$ (mm)	166	104
${\overset{ˇ}{d}}_{\max}$ (mm)	129	101

**Table 3:** The average and the maximum distance between the true TN position and its estimates with (${\overset{ˇ}{d}}_{\mathrm{avg}}$, ${\overset{ˇ}{d}}_{\max}$) and without (${\hat{d}}_{\mathrm{avg}}$, ${\hat{d}}_{\max}$) the use of the statistical model, when using the CI algorithm and the TSML algorithm, are shown.

Scenario 2	CI Algorithm	TSML Algorithm
${\hat{d}}_{\mathrm{avg}}$ (mm)	153	150
${\overset{ˇ}{d}}_{\mathrm{avg}}$ (mm)	62	68
${\hat{d}}_{\max}$ (mm)	229	210
${\overset{ˇ}{d}}_{\max}$ (mm)	130	112

**Table 4:** Values of ${{\left[\hat{d}\right]}_{j}}^{\mathrm{th}}$, expressed in mm, such that $P({\left[\hat{d}\right]}_{j}<{{\left[\hat{d}\right]}_{j}}^{\mathrm{th}})=0.9$ (columns 1, 4, 7, 10); values of ${{\left[\overset{ˇ}{d}\right]}_{j}}^{\mathrm{th}}$, expressed in mm, such that $P({\left[\overset{ˇ}{d}\right]}_{j}<{{\left[\overset{ˇ}{d}\right]}_{j}}^{\mathrm{th}})=0.9$ (columns 2, 5, 8, 11); and their differences Δ= (columns 3, 6, 9, 12) are shown for different scenarios and algorithms.

Good Scenario						Bad Scenario					
CI			TSML			CI			TSML		
${{\left[\hat{d}\right]}_{j}}^{\mathrm{th}}$	${{\left[\overset{ˇ}{d}\right]}_{j}}^{\mathrm{th}}$	Δ	${{\left[\hat{d}\right]}_{j}}^{\mathrm{th}}$	${{\left[\overset{ˇ}{d}\right]}_{j}}^{\mathrm{th}}$	Δ	${{\left[\hat{d}\right]}_{j}}^{\mathrm{th}}$	${{\left[\overset{ˇ}{d}\right]}_{j}}^{\mathrm{th}}$	Δ	${{\left[\hat{d}\right]}_{j}}^{\mathrm{th}}$	${{\left[\overset{ˇ}{d}\right]}_{j}}^{\mathrm{th}}$	Δ
118	39	79	54	35	19	168	74	94	164	81	83

## Abbreviations

The following abbreviations are used in this manuscript:

UWB	Ultra Wide Band
ToF	Time of Flight
FCC	Federal Communications Commission
WSN	Wireless Sensor Network
RSS	Received Signal Strength
CI	Circumference Intersection
TSML	Two-Stage Maximum-Likelihood
